# PSMA PET/CT Kinetics During Systemic Therapy and Radiotherapy in Hormone-sensitive Prostate Cancer: A Systematic Review

**DOI:** 10.1016/j.euros.2026.09.003

**Published:** 2026-09-21

**Authors:** Jennifer Le Guévelou, Matteo Bauckneht, Timo F.W. Soeterik, Matthijs Scheltema, Lorenzo Bianchi, Claudia Kesch, Giorgio Gandaglia, Pawel Rajwa, Rossella Nicoletti, Fabio Zattoni, Alberto Martini, Vittorio Fasulo, Veeru Kasivisvanathan, Felix Preisser, Lara Rodriguez Sanchez, Francesca Ambrosini, Ugo Falagario, Tamas Fazekas, Alexander Giesen, Guido Rovera, Martina Maggi, Chiara Mercinelli, Marcin Miszczyk, Juan Gomez Rivas, Olivier Windisch, Hein Stroomberg, Bernardino Rui, Ali Bourgi, Derya Tilki, Giancarlo Marra, EAU-YAU Prostate Cancer Working Group

**Affiliations:** a Department of Radiation Oncology, Centre Hospitalier Régional Universitaire de Tours, Tours, France; b Nuclear Medicine, IRCCS Azienda Ospedaliera Metropolitana, Genova, Italy; c Department of Health Sciences (DISSAL), University of Genova, Italy; d Department of Radiation Oncology, University Medical Center Utrecht, Utrecht, The Netherlands; e St. Vincent’s Prostate Cancer Research Centre, Sydney, New South Wales, Australia; f Division of Urology, IRCCS Azienda Ospedaliero-Universitaria di Bologna, Bologna, Italy; g Università degli Studi di Bologna, Bologna, Italy; h Department of Urology, West German Cancer Center, University Hospital Essen, Essen, Germany; i Unit of Urology/Division of Oncology, IRCCS Ospedale San Raffaele, Milan, Italy; j Vita-Salute San Raffaele University, Milan, Italy; k Department of Urology, Medical University of Silesia, Zabrze, Poland; l Department of Experimental and Clinical Biomedical Science, University of Florence, Florence, Italy; m Department of Precision Medicine in the Medical, Surgical and Critical Care Area (Me.Pre.C.C.), University of Palermo, Palermo, Italy; n Department of Surgery, Oncology and Gastroenterology (DISCOG), University of Padua, Padua, Italy; o Department of Urology, University of Cincinnati College of Medicine, Cincinnati, USA; p Department of Urology, IRCCS Humanitas Research Hospital, Milan, Italy; q Centre for Urology Imaging, Prostate, AI and Surgical Studies (COMPASS) Research Group, Division of Surgery and Interventional Science, University College London, London, UK; r Department of Urology, Comprehensive Cancer Center, Medical University of Vienna, Vienna, Austria; s Martini-Klinik Prostate Cancer Centre, University Hospital Hamburg-Eppendorf, Hamburg, Germany; t Department of Urology, Institut Mutualiste Montsouris, Paris, France; u Department of Urology, IRCCS Ospedale Policlinico San Martino, Genova, Italy; v Department of Urology and Kidney Transplantation, University of Foggia, Foggia, Italy; w Department of Urology, Semmelweis University, Budapest, Hungary; x Department of Urology, UZ Leuven, Leuven, Belgium; y Department of Development and Regeneration, KU Leuven, Leuven, Belgium; z Department of Nuclear Medicine, San Giovanni Battista Hospital, Città della Salute e della Scienza, Italy; aa Department of Urology, ASST Santi Paolo E Carlo, University of Milan, Milan, Italy; bb Medical Oncology Department, IRCCS Ospedale San Raffaele, Comprehensive Cancer Center, Milan, Italy; cc Università Vita-Salute San Raffaele, Milan, Italy; dd Department of Urology, Comprehensive Cancer Center, Medical University of Vienna, Vienna, Austria; ee Collegium Medicum, Faculty of Medicine, WSB University, Dąbrowa Górnicza, Poland; ff Servicio de Urología, Hospital Clínico San Carlos, Madrid, Spain; gg Universidad Complutense de Madrid, Facultad de Medicina, Departamento de Cirugía, Sección de Urología, Madrid, Spain; hh Department of Urology, University Hospital of Geneva, Geneva, Switzerland; ii Faculty of Medicine, Geneva University, Geneva, Switzerland; jj Urological Research Unit, Department of Urology, Copenhagen University Hospital - Rigshospitalet, Copenhagen, Denmark; kk Biotech Innovation & Research Centre, University of Copenhagen, Copenhagen, Denmark; ll Glickman Urological Institute, Cleveland Clinic, Cleveland, USA; mm Department of Urology, CHRU Tours, Tours, France; nn Martini-Klinik Prostate Cancer Center, University Hospital Hamburg-Eppendorf, Hamburg, Germany; oo Department of Urology, University Hospital Hamburg-Eppendorf, Hamburg, Germany; pp Department of Urology, Koc University Hospital, Istanbul, Turkey; qq Department of Urology, San Giovanni Battista Hospital, Città della Salute e della Scienza and University of Turin, Turin, Italy

**Keywords:** PSMA expression, Hormone-sensitive prostate cancer, Androgen deprivation therapy, Radiotherapy, Positron emission tomography/computed tomography

## Abstract

**Context:**

Although prostate-specific membrane antigen (PSMA) positron emission tomography/computed tomography (PET/CT) is commonly used for both initial staging and restaging at biochemical recurrence for patients diagnosed with prostate cancer (PCa), its role in the evaluation of treatment response is currently not validated.

**Objectives:**

We aimed to identify studies that implemented PSMA PET/CT as a restaging modality for patients with hormone-sensitive PCa (HSPC) and highlight the evolution of PSMA PET/CT parameters to assess its potential use for treatment response evaluation.

**Evidence Acquisition:**

A systematic review was performed using the MEDLINE, Scopus, and Embase databases until December 2025. Studies deemed eligible were those reporting PSMA expression dynamics under either systemic therapy or after radiotherapy (RT).

**Evidence synthesis:**

A total of 11 studies were included, including two prospective single-arm studies and nine retrospective studies. Under androgen deprivation therapy (ADT), a decrease in PSMA expression was observed across all disease sites. In one prospective study, a flare phenomenon (increase ≥20% in tracer uptake) was observed at the lesion level in 26% of bone lesions and 11% of nodal and prostate lesions at 3–4 wk, with a median change in maximum standardized uptake value (ΔSUVmax) of +50% (interquartile range 32–72) in bone lesions showing flare. At 22 wk, one retrospective study reported mean SUVmax reductions of 48%, 59%, and 74% in prostate, nodal, and bone lesions, respectively. Other studies at different time points each reported reductions in prostate SUVmax ranging from 49% to 67%, with differences in whether estimates were summarized as means or medians. After RT, one retrospective study suggested that the reduction in prostate SUVmax may be more pronounced in patients receiving concomitant ADT (mean reduction –59% ± 25% vs –45% ± 31% for RT alone; *p* = 0.004).

**Conclusions:**

PSMA PET-derived parameters hold the potential to personalize treatment strategies depending on treatment response. The relationship between changes in PSMA expression and oncological outcomes warrants further investigation in other studies.

## Take Home Message

PSMA PET-derived volumetric data hold the potential to personalize treatment strategies depending on treatment response. Comprehensive data on PSMA expression dynamics, however, are still needed before this approach can be implemented in routine clinical practice.

ADVANCING PRACTICE **What does this study add?**

PSMA PET/CT is increasingly used for staging and at the time of biochemical relapse; however, it remains unvalidated for the follow-up of patients. This study provides data on the impact of both systemic treatment (ADT) and radiotherapy on PSMA expression and provides insight for the development of new criteria in order to assess treatment response.

**Clinical Relevance**

Changes in PSMA expression on serial PSMA-PET/CT may provide an imaging-based measure of treatment response in men with hormone-sensitive prostate cancer. Incorporating longitudinal PSMA-PET/CT parameters could potentially help identify equivalent patients with different treatment responses and support more individualized treatment strategies. Prospective studies linking changes in PSMA expression to clinical outcomes are needed before PSMA-PET/CT can be routinely used for treatment-response assessment. Associate Editor: Roderick C.N. van den Bergh.

**Patient Summary**

For patients diagnosed with prostate cancer, prostate-specific membrane antigen (PSMA) positron emission tomography/computed tomography (PET/CT) is an accepted imaging strategy to assess the extent of disease at the time of diagnosis and at biochemical relapse. However, PSMA PET/CT is not validated for patient follow-up. Its implementation could unveil new strategies, such as treatment intensification in poor responders, and de-escalation in patients with favorable evolution. Before implementing this imaging strategy in follow-up, more data on PSMA expression changes during systemic therapy or after local treatments are needed.

## 1 Introduction

Computed tomography (CT) combined with bone scintigraphy long remained the standard of care (SOC) for staging men presenting with prostate cancer (PCa) [1]. In the past decade, the ProPSMA trial demonstrated higher accuracy with prostate-specific membrane antigen (PSMA) positron emission tomography (PET)/CT compared with conventional imaging in the detection of metastatic disease [2], [3], leading to its implementation within guidelines as an initial staging modality for patients presenting with unfavorable intermediate- and high-risk PCa [1]. In parallel, further clinical trials established its role as a restaging modality in patients with biochemical recurrence [4], [5]. Yet, to date, conventional imaging remains implemented as a follow-up modality for men with both metastatic hormone-sensitive PCa (mHSPC) and metastatic castration-resistant PCa (mCRPC).

Although the use of PSMA PET/CT as a follow-up modality in the metastatic PCa setting remains largely underassessed, it has the potential to unveil new treatment strategies, as ongoing trials are increasingly implementing personalized strategies for both localized and metastatic PCa. An early prostate specific antigen (PSA) decrease (<0.2 ng/ml [6] or even <0.02 ng/ml at 3–6 mo [7]) has been demonstrated to be a strong prognostic marker for overall survival (OS) [8], thereby paving the way for de-escalated strategies [9]. For patients with poor PSA responses at 6 mo, treatment intensification is currently being evaluated—for instance, PSMA-targeted radioligand therapy (RLT) within the PEACE-6 poor responders phase 3 trial (NCT06496581). Although treatment (de-)escalation using these strategies holds the potential to improve both oncological outcomes and quality of life in patients with PCa, treatment personalization based solely on biological parameters may miss a window of opportunity for local ablative approaches performed either for oligopersistent or oligoprogressive lesions. Aside from better visualization of tumor sites, metrics such as PSMA expression kinetics can be analyzed. PSMA expression kinetics describe temporal changes in quantitative PSMA PET parameters (standardized uptake value (SUV)max, SUVpeak, and metabolic tumor volume [MTV]), with the aim to estimate variations in tumor PSMA avidity and burden over time. Recently, PSMA PET parameters have been suggested to be reliable parameters for treatment response and survival, both in the hormone-sensitive and castration-resistant settings [10], [11]. As a result, implementing PSMA PET/CT for the follow-up of patients with HSPC might hold the potential to both predict outcomes and propose early (de-)escalation strategies. Several challenges persist before PSMA PET/CT-based strategies can be implemented in trials for treatment personalization in patients with HSPC, most notably the near-complete lack of data regarding the evolution of PSMA expression under systemic therapy and after radiotherapy (RT).

We aimed to report data on studies that implemented PSMA PET/CT as a restaging modality for patients with HSPC. Ultimately, we aimed to shed light on perspectives on treatment personalization unveiled by the implementation of PSMA PET/CT for the assessment of treatment response in patients with HSPC.

## 2 Evidence acquisition

### 2.1 Data sources and searches

This systematic review was reported according to the Preferred Reporting Items for Systematic Reviews and Meta-Analyses guidelines (PRISMA) [12]. The protocol was registered in the International Prospective Register of Systematic Reviews (PROSPERO) database (*CRD420251246709*). A systematic literature search was conducted in December 2025 using three databases (MEDLINE through PubMed, Embase, and Scopus). The full search strategy is available in Supplementary Table 1.

### 2.2 Study selection

Records were screened for relevance based on the Population/Concept/Context framework:

1. Population (P): adult patients with localized, locally advanced, or metastatic (either de novo or oligorecurrent) HSPC who underwent PSMA PET/CT at baseline and at least one follow-up PSMA PET/CT during or after treatment.
2. Concept (C): the evolution of quantitative PSMA PET/CT parameters (primarily SUVmax, but also SUVmean, PSMA-derived tumor volume, and total lesion PSMA where reported) under systemic therapy (androgen deprivation therapy [ADT] ± androgen receptor pathway inhibitor (ARPI) or chemotherapy) or after RT, compared with baseline.
3. Context (Co): serial PSMA PET/CT assessment during or after systemic therapy, rRT, or metastasis-directed therapy (MDT) in HSPC.

For inclusion criteria (publication eligibility), peer-reviewed clinical studies, irrespective of design, were eligible; preclinical studies, case reports, editorials, letters, comments, and reviews were excluded, as were studies with fewer than ten patients.

All preclinical studies, case reports, editorials, letters, comments, as well as reviews were excluded. The following were also not deemed eligible: studies with fewer than ten patients, studies reporting data on both castration-resistant and HSPC with no specific data provided on both populations. All abstracts and full texts were screened by two different reviewers (J.L.G. and T.F.). The extraction process was performed by the two reviewers independently. Discrepancies were resolved with the help of a third reviewer (M.M.), who independently performed the extraction process, followed by a discussion with the first two reviewers. The selection process is summarized in the PRISMA flowchart (Fig. 1).

**Fig. 1:**
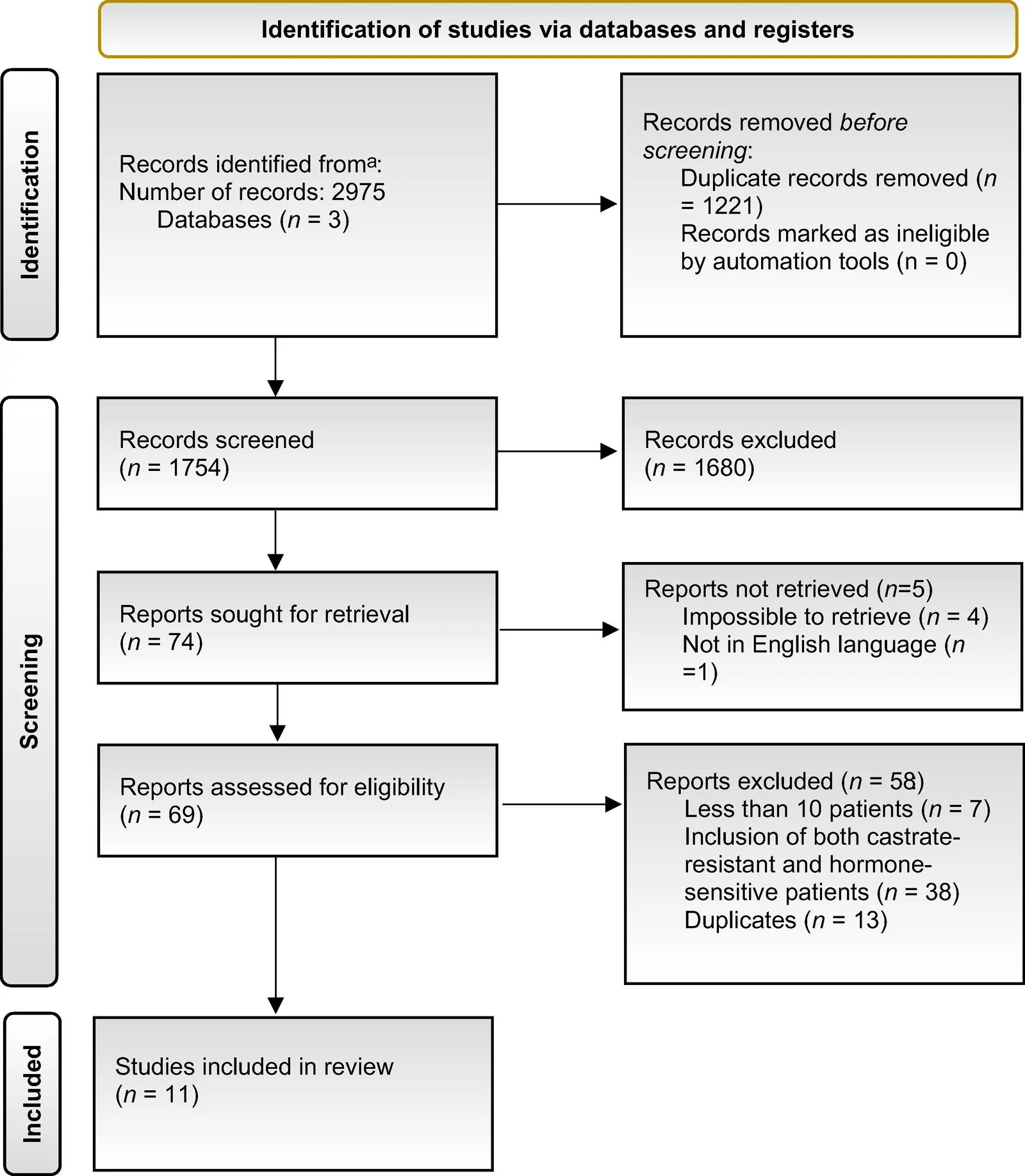
PRISMA flowchart. PRISMA = Preferred Reporting Items for Systematic Reviews and Meta-Analyses; PSMA = XXX. ^a^ Exclusion of studies testing PSMA in the castration-resistant setting or in other types of cancer; preclinical studies, case reports, editorials, letters, comments, reviews.

### 2.3 Risk of bias assessment

The methodological quality of the included studies was assessed using a modified version of the JBI critical appraisal checklist for case series. The standard Joanna Briggs Institute (JBI) case series checklist does not categorize domains or overall study quality as low, moderate, or serious risk of bias. Accordingly, a modified approach inspired by the Quality in Prognosis Studies (QUIPS) tool was applied, in which each appraised domain was mapped to a three-level risk-of-bias category: low, moderate, or serious. The following domains were assessed: (1) clarity of inclusion criteria, (2) standardized and reliable measurement of the condition, (3) consecutive or complete inclusion of participants, (4) adequacy of reporting of clinical and demographic information, (5) completeness of outcome reporting, and (6) appropriateness of statistical analysis. An overall risk-of-bias rating was assigned for each study as the worst-rated domain. The detailed item-by-item judgments for each JBI item in each study, the item-to-domain mapping, and the conversion rules used are provided in the Supplementary Methods (Supplementary File 1).

### 2.4 Data synthesis

The primary aim of this review was to report the evolution of PSMA expression (assessed as change from baseline) under systemic therapy and after RT. Secondary aims included the following: (1) the correlation between changes in PSMA PET/CT parameters over time and concurrent changes in serum PSA levels and (2) the prognostic value of the magnitude or direction of PSMA PET/CT parameter change (ie, the extent of SUVmax reduction or the occurrence of a flare phenomenon) for clinical outcomes such as progression-free survival and biochemical recurrence-free survival. Considering the substantial clinical and methodological heterogeneity across included studies—including differences in disease stage, treatment regimens, radiotracers used, and timing of post-treatment imaging—a meta-analysis could not be performed. A narrative synthesis was performed, analyzing the evolution of PSMA expression under systemic therapy (ADT, ARPI, and chemotherapy) separately and after RT. Data were also synthesized depending on the tumor site (primary prostate, nodal, and bone disease).

## 3 Evidence synthesis

### 3.1 Study selection and characteristics

This search retrieved 2975 records; after removal of 1221 duplicates, 1754 records were screened. Of the 74 full-text reports retrieved, 69 were assessed for eligibility (five could not be obtained). After full-text reading, 11 studies were included, including two prospective single-arm studies and nine retrospective studies. Six studies reported PSMA expression under systemic therapy consisting of ADT with or without androgen receptor pathway inhibitors (one study) or docetaxel (one study); five provided results after RT (either on the prostate or on secondary lesions). Among the included studies, all reported outcomes at the patient level with the exception of Malaspina et al. [13], which reported PSMA PET/CT parameter changes at the lesion level. Notably, some studies included patients who received different types of ADT, and some studies reported overall variations in PSMA PET/CT parameters without clearly describing variations separately in patients who did or did not receive ADT; these represent sources of clinical and methodological heterogeneity and limit direct cross-study comparisons. Furthermore, some studies reported outcomes at different time points, adding further methodological heterogeneity.

### 3.2 Risk of bias

According to the modified JBI case series checklist, studies were assessed as having a low risk of bias (one study), a moderate risk of bias (five studies), and a serious risk of bias (five studies). The risk of bias related to inclusion criteria was low across all studies, and the condition was measured in a standardized and reliable way in most participants. Three studies were assessed as having a serious risk of bias because of incomplete inclusion of participants. Moderate or serious risk of bias related to the reporting of clinical and demographic information was noted in five studies. The most important source of bias concerned the reporting of outcomes, where several studies reported only absolute values for changes in PSMA parameters without providing percentages, measures of variability (range, interquartile range [IQR], or standard deviation (SD)), or subgroup comparisons (Table 1) [13], [14], [15], [16], [17], [18], [19], [20], [21], [22], [23]. Of note, variability in treatment type (eg, type of ADT and RT dose), disease stage, and imaging timing across studies constitutes clinical and methodological heterogeneity rather than bias per se and should be interpreted as such when comparing results across studies.

**Table 1:** Risk of bias assessment using a modified JBI case series checklist. Each domain and the overall study quality are categorized as low, moderate, or serious risk of bias based on an adaptation of the standard JBI case series checklist items to the specific appraisal domains listed below.

**Study**	**Year**	**Inclusion criteria**	**Measurement of the condition**	**Inclusion of the participants**	**Reporting of clinical and demographic information**	**Reporting of outcomes**	**Statistical analysis**	**Overall results**
Malaspina et al. [13]	2023	Low	Low	Low	Low	Low	Low	Low
Tseng et al. [14]	2022	Low	Low	Moderate	Low	Moderate	Low	Moderate
Hoberück et al. [15]	2020	Low	Moderate	Serious	Moderate	Moderate	Moderate	Serious
Afshar-Oromieh et al. [16]	2018	Low	Moderate	Serious	Moderate	Moderate	Low	Serious
Onal et al. [18]	2020	Low	Low	Moderate	Low	Moderate	Low	Moderate
Onal et al. [17]	2023	Low	Low	Moderate	Low	Moderate	Moderate	Moderate
Onal et al. [23]	2024	Low	Moderate	Moderate	Low	Moderate	Low	Moderate
Sheikh et al. [19]	2024	Low	Moderate	Serious	Moderate	Moderate	Low	Serious
Zang et al. [20]	2025	Low	Low	Moderate	Serious	Moderate	Moderate	Serious
Gawish et al. [22]	2023	Low	Moderate	Moderate	Moderate	Moderate	Moderate	Moderate
Sadetski et al. [21]	2021	Low	Moderate	Moderate	Low	Moderate	Serious	Serious

JBI = Joanna Briggs Institute.

Note on risk-of-bias tool: The standard JBI critical appraisal checklist for case series does not produce low/moderate/serious ratings. The ratings in this table were derived using a modified version of the JBI case series checklist in which each appraised item was mapped to a three-level risk-of-bias category (low, moderate, and serious) following Quality in Prognosis Studies (QUIPS)-inspired guidance. Domains assessed were: (1) clarity of inclusion criteria, (2) standardized and reliable measurement of the condition, (3) consecutive/complete inclusion of participants, (4) reporting of clinical and demographic information, (5) reporting of outcomes, and (6) appropriateness of statistical analysis.

Detailed item-by-item judgments, the item-to-domain mapping, and rating rules are provided in the Supplementary Methods (Supplementary File 1).

### 3.3 Evolution of PSMA expression under systemic treatment

A limited number of studies assessed the modulation of PSMA expression in both primary and metastatic lesions under systemic therapy, with most of the data regarding patients with HSPC receiving ADT (Table 2) [13], [14], [15], [16], [17], [18], [19], [20], [21], [23], [24]. Only one study included patients who received ADT with ARPI (13% of the patients) [14], and one study included patients who received docetaxel (24%) [15]. However, these studies do not report the evolution of PSMA parameters in this population of patients.

**Table 2:** Studies reporting the evolution of PSMA PET/CT parameters during systemic treatment or after RT in HSPC

**Study**	**Design**	***n***	**Disease setting**	**Treatment**	**Tracer**	**Median time of** **assessment(wk)**	**Unit ofanalysis**	**Evolution of PSMA PET/CT parameters(main result)**	**Prognostic PET/CT parameters(numerical estimates)**
**Systemic treatment**									
Malaspina et al. [13]	Prospective	25	De novo mHSPC	ADT(GnRH antagonist)	18F-PSMA-1007	3–4	Lesion-level (57 prostate/ 314 LN/406 bone lesions)	Lesions with flare (increase ≥20%): Prostate: 11% of lesions Median ΔSUVmax +45% (IQR 20–106) Node: 11% of lesions Median ΔSUVmax +60% (IQR 32–114) Bone: 26% of lesions Median ΔSUVmax +50% (IQR 32–72) Lesions with decrease/no change: Prostate 89%: median ΔSUVmax –28% (IQR –45 to –7) Node 89%: median ΔSUVmax –51% (IQR –74 to –21) Bone 74%: median ΔSUVmax –33% (IQR –53 to –15)	Negative correlation between ΔSUVmax and PSA change under ADT (*p* < 0.001)
Tseng et al. [14]	Prospective	30	Locally advanced orde novo mHSPC	ADT ± ARPI(13% of patients)	68Ga-PSMA-11	10–14	Patient-level	Mean change in SUVmax (± SD): Prostate: –49% ± 52% Node: –62% ± 37% Bone: –63% ± 34%Response (mPERCIST 1.0): PR 80%, CR 6.7%, SD 6.7%, PD 6.7%	Less prominent mean SUVmax reduction in ISUP grade 5 versus <5 (wbSUVmax reduction ratio 0.35 vs 0.60; *p* = 0.006)
Onal et al. [18]	Retrospective	108	Localized or locallyadvanced HSPC	Neoadjuvant ADT	68Ga-PSMA-11	12	Patient-level	Median change in prostate SUVmax: –66% (range 5–100%)Decrease in 84% of patients No change or increase in 16% (median +24%, range 0–198%)	Higher median SUVmax reduction in GS 7 versus GS >7 Less prominent reduction in ISUP grade 5 pattern
Hoberück et al. [15]	Retrospective	21	De novo oligometastaticHSPC	ADT ± docetaxel(24% of patients)	68Ga-PSMA-11	22	Patient-level (109 lesions alsoanalyzed)	Mean change in SUVmax: Prostate: –48% (range/IQR: NR) Node: –59% (range/IQR: NR) Bone: –74% (range/IQR: NR)miN-/miM-downstaging in 52.7% of patients	Modest correlation between residual TL-PSMA and PSA at T2 (*r* = 0.504; *p* = 0.020) Intraprostatic tumor volume versus PSA at T2: (*r* = 0.456; *p* = 0.038)
Onal et al. [17]	Retrospective	71	Localized PCa(high-risk)	Neoadjuvant ADT	68Ga-PSMA-11	12	Patient-level	Median change in prostate SUVmax: –67% (range 2–100%) Decrease in 91% of patientsIncrease in 10% (median +54%, range 4–98%)	Higher SUVmax response rate in GS 7 versus GS >7 (59% vs 40%; *p* = 0.04) SUVmax change predictive of bRFS (CR/PR vs SD/PD: 66% ± 22% vs 1% ± 12%; *p* < 0.001)
Afshar-Oromieh et al. [16]	Retrospective	10	De novo PCa(castration-sensitive)	ADT (GnRH agonist/anti-androgen/both)	68Ga-PSMA-11	33(range 42–369 d)	Lesion-level (31 lesions;10 patients)	Mean change in prostate SUVmax: –56% (range/IQR: NR; *p* < 0.0001)SUVmax flare (increase) in 13% of patients (16.1% of lesions) Lesion visibility: 31 → 14 lesions during ADT Lesion number decrease: *p* = 0.0199	SUVmax decrease *p* < 0.0001 Tumor volume decrease *p* = 0.0015 Lymph node lesions more likely to disappear than bone (*p* = 0.0499) Median follow-up: 33 wk (range 6–53)
**RT/metastasis-directed therapy**									
Sheikh et al. [19]	Retrospective	32	Oligorecurrent HSPC(bone metastases)	MDT (RT)± concurrent ADT	68Ga-PSMA-11or 18F-PSMA-1007	13 (post-RT) 52 weeks (follow-up)[see note†]	Lesion-level (49 bone lesions;32 patients)	Overall: median change in SUVmax –60%(range –98% to +54%; 94% of lesions decreased) Median absolute change: –3 (range –82 to 0) By ADT use (patient-level, absolute SUVmax change): With ADT: median –12 (range –82 to 0) Without ADT: median –3 (range –7 to +2)	Median time to PSMA nadir: 13 mo (range 4–63) 5-yr PFS reported: NR by patient subgroupPSA decline ≥50% in 56% of patients at first FU
Sadetski et al. [21]	Retrospective	12	Oligorecurrent HSPC	MDT alone (SBRT)	68Ga-PSMA-11	68	Lesion-level (15 bone lesions;12 patients)	Median change in bone SUVmax: –80%(pre: median 6 [range 2–13] → post: median 1) Complete metabolic response: 14/15 lesions (93%) Patient-level: 5/12 (42%) remained free of new metastases at 2 yr; 5/12 developed ≤3 new oligometastases; 2/12progressed to widespread disease	Local control: 93% during follow-up No in-field recurrences PSA nadir: median 0.01 ng/ml post-treatment(median time to nadir 7 mo)
Zang et al. [20]	Retrospective	48	De novo oligometastaticor oligorecurrent HSPC	MDT (SABR 35 Gy/5 fractions)± ADT	68Ga-PSMA-11	13 (1st FU) → nadir at 19 mo[see note‡]	Lesion-level (97 lesions: 67 bone + 30 LN;48 patients)	At 1st FU (median 13 mo): Median change in SUVmax: –80% (pre: median 11 [range 2–122] → post: median 2 [0–26]) CR: 49 lesions (51%) at 1st FU; 90% by end of follow-up Median time to SUVmax nadir: 19 mo (range 3–50)5-yr local control: 86% (95% CI 76–97%)	5-yr local control: 86% (95% CI 76–97%) Median PFS reported: NRMedian OS reported: NR
Gawish et al. [24]	Retrospective	17	Oligorecurrent HSPC	MDT (IGRT/SBRT)	68Ga-PSMA-11	32(range 6–36 mo)	Lesion-level (28 lesions: 21 LN + 7 bone;17 patients)	Median change in SUVmax: –87% (pre: median 8 [range 3–58] → post: median 1 [0–58]; *p* < 0.001) Complete metabolic response: 27/28 lesions (96%)1 lesion (LN) showed an increase (38→58)	Local metabolic control: 96% at 1 yr Local control (BED_10_ >67 Gy): 100% Median follow-up: 17 mo (6–36) Overall survival: 100% during follow-up No grade 3–4 toxicity
Onal et al. [23]	Retrospective	71	Localized PCa(intermediate-risk)	Definitive RT ± ADT (78 Gy/39 fx prostate;86 Gy focal boost)	68Ga-PSMA-11	28	Patient-level (primary prostatetumor)	Median change in prostate SUVmax: –61.2% (range/IQR: NR) Decrease in 93% of patients Mean change in SUVmax ± SD: RT alone: –45% ± 31% RT + ADT: –59% ± 25% (*p* = 0.004)	Less pronounced mean SUVmax reduction with RT alone versus RT + ADT (–45% ± 31% vs –59% ± 25%; *p* = 0.004) No significant correlation betweenΔSUVmax and ΔPSA

PSMA = prostate -specific membrane antigen; PET/CT = positron emission tomography/computed tomography; mHSPC = metastatic hormone-sensitive prostate cancer; HSPC = hormone-sensitive prostate cancer; PCa = prostate cancer; ADT = androgen deprivation therapy; ARPI = androgen receptor pathway inhibitor; GnRH = gonadotropin-releasing hormone; MDT = metastasis-directed therapy; RT = radiotherapy; SBRT/SABR = stereotactic body/ablative body radiotherapy; IGRT = image-guided radiotherapy; GS = Gleason score; ISUP = International Society of Urological Pathology; SUVmax = maximum standardized uptake value; ΔSUVmax = change in SUVmax; TL-PSMA = total lesion PSMA; CR = complete response; PR = partial response; SD = stable disease; PD = progressive disease; bRFS = biochemical recurrence-free survival; OS = overall survival; LN = lymph node; IQR = interquartile range; NR = not reported; Values after ± denote standard deviation (SD); BED_10_ = biologically effective dose (α/β = 10); PFS = progression free survival; PSA = prostate specific antigen; mPERCIST = modified Positron Emission Tomography Response Criteria in Solid Tumors; FU = follow up.

† Multiple time points of assessment: Sheikh et al. [19] performed PSMA PET/CT at a median of 13 mo post-RT (first follow-up) and at a median of 52 wk (overall follow-up); the SUVmax change of –60.4% refers to the lesion-level result at the post-RT assessment. Zang et al. [20] performed a first follow-up PET/CT at a median of 13 mo after SABR, with the SUVmax nadir reached at a median of 19 mo; the 5-yr local control figure is reported at maximum follow-up.

‡ Two time points reported in the same study: Sheikh et al. [21] report SUVmax change at 13 wk post-RT and median follow-up at 52 wk; Zang et al. [20] report SUVmax change at first follow-up (median 13 mo) and time to nadir (median 19 mo). ± values denote standard deviation (SD. All values are expressed as mean ± SD or median (range/IQR) as reported in the source studies. Units of analysis are reported per row: patient-level = one value per patient; lesion-level = one value per individual metastatic site. Percentages are rounded to the nearest whole number per the journal’s precision guidelines.

Malaspina et al. [13] reported the evolution of PSMA expression at the lesion level in 25 patients presenting with de novo mHSPC, treated within a prospective study at 3–4 wk after ADT initiation. This study used a lesion-level rather than patient-level unit of analysis, which should be noted when interpreting these results alongside patient-level data from other studies. In lesions not showing a flare, the median decrease in prostate SUVmax was –28% (IQR –45 to –7). After 12 wk of ADT, a median reduction in prostate SUVmax of –67% (range 2–100%) was reported by Onal et al. [18] and Onal et al. [17], and a mean reduction of –49% ± 52% was reported by Tseng et al. [14]. In the study by Hoberück et al. [15] the mean reduction in prostate SUVmax after 22 wk was –48% (range/IQR not reported). Finally, Afshar-Oromieh et al. [16] reported a mean decrease in prostate SUVmax of –56% (range/IQR not reported) after a mean ADT duration of 33 wk. Although most prostate lesions tend to show a decreased expression of PSMA under ADT, a flare phenomenon (ie, an increase in PSMA expression despite PSA response) was described at the lesion level in 11% of prostate lesions (Malaspina et al. [13]) and in 13% of the patients (Afshar-Oromieh et al. [16]) in the first few months of ADT. Of note, ADT type was clinically heterogeneous across studies. In the study by Malaspina et al. [13], all patients received a Gonadotropin-releasing hormone (GnRH) antagonist, whereas in the study by Afshar-Oromieh et al. [16], patients received either a GnRH agonist, a nonsteroidal anti-androgen, or both. The heterogeneity in ADT type and duration (range 42–369 d in Afshar-Oromieh et al. [16]) represents a source of clinical heterogeneity that limits direct comparisons across studies.

Only three studies reported data on the evolution of PSMA expression within nodal and bone lesions under ADT (Table 2). A decrease in PSMA expression was observed across all disease sites, with a numerically more marked reduction in bone compared with nodal lesions: Hoberück et al. [15] reported a mean reduction in bone SUVmax of –74% at 22 wk (vs –59% for nodal lesions; range/IQR not reported). A flare phenomenon has been reported most frequently at the lesion level in bone metastases within the first 1 mo after ADT initiation: Malaspina et al. [13] reported a flare in 26% of bone lesions (median ΔSUVmax +50%, IQR 32–72) compared with 11% of nodal lesions (median ΔSUVmax +60%, IQR 32–114) and 11% of prostate lesions (median ΔSUVmax +45%, IQR 20–106). These results derive from a single prospective study and should not be generalized without further confirmatory evidence.

Discrepancies exist between studies regarding a potential link between PSMA expression and biochemical response. Within the prospective study led by Malaspina et al. [13], a linear and negative correlation was found between variation in SUVmax and PSA levels (correlation coefficient not reported; *p* < 0.001). However, in the retrospective study led by Hoberück et al. [15], although all PSMA parameters appeared to correlate with each other, total molecular tumor volume (total lesion (TL)-PSMA) showed only a modest correlation with biochemical response (*r* = 0.50; *p* = 0.020).

### 3.4 Evolution of PSMA expression following RT

Scarce data are available regarding PSMA expression kinetics following nodal or bone MDT for oligometastatic HSPC. Sheikh et al. [19] reported outcomes after MDT for bone lesions in patients with oligorecurrent HSPC. Biologically effective dose (α/β = 3) ranged between 66 Gy and 93 Gy. The wide variation in RT dose delivered represents a source of clinical and methodological heterogeneity that limits interpretation of the impact of RT dose on SUVmax evolution, contributing to the serious risk of bias rating for this study. After a median follow-up of 13 mo, the median relative change in SUVmax was –60%. Reduction in SUVmax has been suggested to vary depending on whether ADT was used, with a median SUVmax change at 13 mo reaching –12 (range –82 to +0.44) and –2.9 (range –7.3 to +1.8), with and without ADT, respectively. Zang et al. [20] reported outcomes after MDT for either nodal or bone lesions, in a retrospective study including 48 patients diagnosed with either de novo oligometastatic or oligorecurrent HSPC. All patients received MDT performed at a total dose of 35 Gy in five fractions. The median time to obtain the nadir of PSMA uptake was 19 mo. Of note, data on nodal and bone lesions were not analyzed separately, which represents a serious risk of bias when interpreting the result, as PSMA PET/CT behavior between nodal and bone lesions might differ. Two studies reported outcomes after MDT alone for oligorecurrent PCa. The longest follow-up was provided in the study led by Sadetski et al. [21] showing at 68 wk a median reduction of SUVmax in bone lesions reaching 80%. Complete response on repeat PSMA PET/CT was observed in 93% of the lesions. Gawish et al. [22] showed, after a median follow-up of 32 wk, a median reduction in SUVmax reaching 87%, in a population of oligorecurrent patients presenting with either nodal (21 relapses) or bone (seven lesions) relapses. As in the study led by Zang et al. [20], the absence of separate reporting of nodal and bone lesion outcomes represents a source of methodological heterogeneity.

Finally, Onal et al. [23] reported PSMA PET/CT parameters after prostate RT with or without ADT for patients diagnosed with intermediate-risk localized PCa. The total doses prescribed for the prostate and focal boost to the intraprostatic lesion were 78 and 86 Gy, respectively, delivered in 39 fractions. At a median follow-up of 28 wk, prostate SUVmax decreased by a median value of 61%. The mean SUVmax reduction was less prominent in patients receiving RT alone (45% ± 31% vs 59% ± 25%; *p* = 0.004). In this population of patients, no significant correlation between SUV changes and PSA changes was observed.

Overall, as studies assessing the evolution of PSMA PET/CT parameters are either associated with moderate or high risk of bias (Table 1), the interpretation of the evolution of PSMA PET/CT parameters after RT should be cautious.

### 3.5 Discussion

Prostate SUV_max_ appears to be halved after a period of ADT ranging from 10–22 wk, with variations potentially attributed to differences in treatment regimen (eg, use or nonuse of docetaxel). PSMA expression appears to decrease markedly in bone, with a mean reduction reaching up to 74% at 22 wk. A negative linear correlation was found between variation in SUVmax and PSA levels (*p* < 0.001), which might suggest the existence of a link between the reduction of PSMA expression and biochemical response. However, this finding was not confirmed in other studies, suggesting that differences might exist depending on the tracer used and the timing of assessment. Discrepancies might also exist between these two parameters (PSMA and PSA response) depending on tumor biology, as patients with an International Society of Urological Pathology (ISUP) grade 5 pattern in biopsy samples have been suggested to show a more modest SUVmax reduction under ADT [14]. Currently, no head-to-head comparison has directly determined which parameter (PSMA PET/CT or PSA response) is associated with the highest accuracy when it comes to prognosis or prediction of treatment response. After MDT, reduction in SUVmax has been suggested to vary depending on whether ADT was used, with a more pronounced reduction observed in patients receiving ADT. These results appear to be hypothesis-generating but are limited by the retrospective design, the small number of patients included across studies, the use of different radiotracers, and heterogeneity in the timing of evaluation. Further studies are required to determine the impact of total dose and type of systemic therapy on PSMA expression after MDT.

Several strategies to personalize treatment based on prognostic criteria are currently implemented within prospective trials. The THUNDER phase 2/3 trial currently tests the addition of darolutamide in patients with high-risk PCa with either PSMA-positive nonlocalized disease or a high-risk Decipher score (≥0.6). Another strategy of treatment intensification based on PSA response is currently proposed in the PEACE-6 poor responder phase 3 trial, testing treatment intensification with Lutetium-177 (^177^Lu)–PSMA-617 in patients with mHSPC with a PSA ≥0.2 ng/ml after 6 mo of systemic treatment. Although early PSA response is predictive of OS in patients with mHSPC receiving ARPI [24], a small proportion of patients (8%) have been shown to experience induced oligoprogression under systemic treatment even in the absence of biochemical progression [25]. Although being more accurate than conventional imaging regarding tumor localization, PSMA PET-derived volumetric data might also be integrated into personalized treatment strategies that include either escalation or de-escalation depending on treatment response. However, as current data are largely based on retrospective and single-arm observational studies, there is currently no proof that PSMA PET/CT-guided escalation or de-escalation strategies will ultimately improve oncological outcomes. In the castration-resistant setting, parameters including delta (d) of standardized uptake value (SUV_mean_, SUV_max_, SUV_peak_)_,_ and dRECIST are also suggested as potential reliable parameters for systemic treatment response monitoring [10]. Notably, a single best PSMA PET/CT parameter for treatment response has not been established. SUVmax is easy to measure and useful for lesion-level assessment, but volumetric parameters such as MTV or total lesion volume are generally more representative of overall disease burden and are increasingly favored for response evaluation. For example, in the hormone-sensitive setting, MTV was shown to exhibit superior prognostic value for castration resistance-free survival compared with other PET/CT-derived parameters, as well as clinical parameters [11]. The “optimal” parameter might therefore depend on the clinical situation and the type of treatment received (whether local or systemic). Personalized strategies depending on PET/CT response parameters are already validated for several types of cancer, including Hodgkin lymphoma (AHL 2011 [26]) and testicular cancer (SAKK 01/10 trial [27]), based on their ability to significantly reduce both acute and late severe toxicity rates while maintaining similar OS. As growing evidence exists on the possibility of predicting OS in patients with mHSPC and mCRPC with the use of PSMA PET/CT-derived scores [28], [29], such scores could be implemented within prospective studies to select patients for escalated strategies including systemic treatments, ^177^Lu-PSMA-617, or MDT (Fig. 2). Last but not least, prostate RT in the metastatic setting has been widely tested in the past decade following the observation that despite a favorable biochemical response, tumor cells remain within the primary site even after aggressive systemic treatment [30]. Although the PEACE-1 trial did not demonstrate any survival benefit of adding prostate RT in an unselected population of patients with mHSPC receiving the SOC (ADT plus docetaxel with or without abiraterone and prednisone) [31], [32], patients with oligopersistent (ie, persistence of a few metastases under systemic treatment, according to the European Society for Radiotherapy and Oncology - European Organisation for Research and Treatment of Cancer (ESTRO-EORTC) consensus [33]) disease within the prostate gland might be the optimal population to benefit from the addition of prostate RT.

**Fig. 2:**
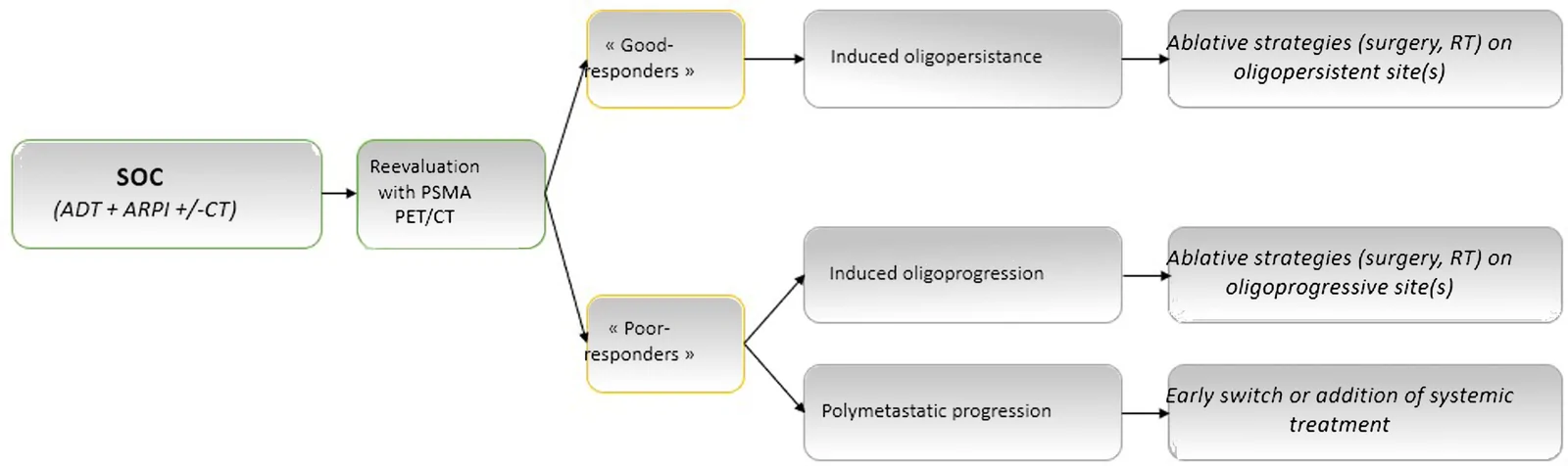
Perspective on treatment personalization for metastatic hormone-sensitive prostate cancer, based on PSMA PET/CT. SOC = standard of care; ADT = androgen deprivation therapy; ARPI = androgen receptor pathway inhibitor; CT = chemotherapy; PSMA = prostate-specific membrane antigen; PET/CT = positron emission tomography/computed tomography; RT = radiotherapy.

PSMA-RLT emerged in the past decade as a treatment modality for mCRPC [34], and more recently for the management of mHSPC. Yet, not much is known regarding the optimal treatment sequence when it comes to the incorporation of PSMA-RLT for patients with mHSPC. Within the phase 2 UpFrontPSMA trial, randomizing ^177^Lu-PSMA-617 and SOC (ADT plus docetaxel) versus SOC alone, ^177^Lu-PSMA-617 was planned to be introduced within the first 1 mo of ADT, followed by docetaxel starting 6 wk after the last dose of ^177^Lu-PSMA-617 [35]. In the PSMAddition phase 3 trial, randomizing ^177^Lu-PSMA-617 plus SOC (ADT and ARPI) or SOC alone, PSMA-RLT was planned to start either simultaneously or in a delayed manner with respect to SOC. Although PSMA upregulation occurring in the first few months of ADT can be challenging when it comes to the assessment of treatment response, it raises the possibility of increasing the dose of radiation delivered to target lesions, thereby potentially increasing the efficacy of PSMA-RLT. Data regarding the kinetics of PSMA PET/CT signal after combined ADT and ARPI and/or chemotherapy are, however, required to further optimize treatment sequencing with our current SOC.

The wide implementation of PSMA PET/CT as a follow-up modality for patients with PCa is currently limited by challenges in discerning flare-ups and dissociated responses in cases of discrepancy between radiological and biochemical assessments. To address this matter, in a consensus statement on PSMA PET/CT response assessment criteria in PCa, Fanti et al. [36] proposed not to perform PSMA PET/CT in the first 3 mo following the introduction of ADT and also advised against its use in cases of biochemical response. Yet, the optimal way to implement this imaging modality has yet to be determined. From a practical perspective, given that PSA kinetics are sufficiently reliable for monitoring treatment response in mHSPC, PSMA PET/CT should ideally be performed at the time of biochemical nadir to establish a reference for subsequent progression. Another challenge is the absence of validated criteria for treatment response, which represents another pitfall to overcome before implementing PSMA PET/CT as a follow-up modality for HSPC, as criteria such as RECIP or PROMISE have, to date, been validated only for patients with mCRPC receiving PSMA-RLT [28], [37]. The consensus statement led by Fanti et al. [36] agreed to categorize as progressive disease either an increase in uptake or tumor volume of >30%. However, the recent SPARC consensus did not validate any response or progression criteria based on PSMA PET/CT [38], highlighting the urgent need for prospective trials to evaluate the robustness of PSMA PET/CT as a monitoring strategy.

This systematic review has some limitations. First, only a limited number of studies have assessed PSMA dynamics under systemic therapy, most of which are of low quality due to their retrospective design. Also, five studies included in this systematic review are associated with a serious risk of bias. More specifically, two studies had bias regarding the measurement of outcomes, as they did not report whether the same radiotracer was used at baseline and for the follow-up measurement. Considering the relatively low number of studies included in this systematic review, the figures listed above may be significantly affected. Bias also exists regarding the selection of patients participating in studies because of either doubts regarding hormone-sensitivity status or the type of treatment received between the two PET/CT scans. As a result, this can significantly affect the applicability of the results to the general population. Also, as no formal within-study comparisons have been performed, it remains difficult to compare the evolution of SUVmax between tumor sites (prostate, bone, and nodes), and differential SUVmax changes should be interpreted with caution. Second, comparisons across studies are hampered by the use of different radiotracers. Some studies reported combined PSMA kinetics for nodal and bone lesions, thereby limiting the interpretability of these findings. Finally, SUVmax is highly sensitive to noise and acquisition parameters and does not reflect total tumor burden, which is particularly relevant in mHSPC. Current PSMA PET literature increasingly favors volumetric parameters such as PSMA MTV and PSMA total lesion, or more robust metrics like SUVpeak, but for now data on kinetics using these parameters are clearly limited. In any case, correlations between PSMA parameters and long-term end points will be needed before implementing them for treatment personalization in either clinical trials or routine practice*.* In the future, large retrospective studies or post hoc analyses coming from prospective studies will be needed to report the evolution of PSMA PET/CT parameters under various types of systemic therapy (including ADT, ARPI, or chemotherapy) and local strategies, and correlate them with long-term patient outcomes. Hopefully, such studies can provide enough data to support the implementation of PSMA PET/CT parameters into personalized prospective trials for patients with HSPC.

## 4 Conclusions

PSMA PET/CT remains unvalidated for the follow-up of patients with PCa. However, PSMA PET/CT parameters such as SUVmax have the potential to personalize treatment strategies depending on treatment response. Volumetric parameters such as PSMA MTV or total lesion PSMA, which have been shown to exhibit superior prognostic value compared with SUVmax, might also be integrated into future studies. Comprehensive data on PSMA expression dynamics, however, are still needed before implementing this strategy in routine clinical practice.

  ***Author contributions:*** Jennifer Le Guévelou had full access to all the data in the study and takes responsibility for the integrity of the data and the accuracy of the data analysis.

  Study concept and design: Le Guévelou, Marra.

Acquisition of data: Le Guévelou, Fazekas, Miszczyk.

Analysis and interpretation of data: None.

Drafting of the manuscript: Le Guévelou.

Critical revision of the manuscript for important intellectual content: All authors.

Statistical analysis: None.

Obtaining funding: None.

Administrative, technical, or material support: None.

Supervision: Marra.

Other: None.

  ***Financial disclosures:*** Jennifer Le Guévelou certifies that all conflicts of interest, including specific financial interests and relationships and affiliations relevant to the subject matter or materials discussed in the manuscript (eg, employment/affiliation, grants or funding, consultancies, honoraria, stock ownership or options, expert testimony, royalties, or patents filed, received, or pending), are the following: None.

  ***Funding/Support and role of the sponsor:*** None.

## Appendix A Supplementary material

Supplementary Data 1 Research strategy - assessment of bias.
