# Supplementary material for: PSMA PET/CT Kinetics During Systemic Therapy and Radiotherapy in Hormone-sensitive Prostate Cancer: A Systematic Review
Source: Eur Urol Open Sci. 2026 Sep 21;93:14–25. doi: 10.1016/j.euros.2026.09.003 (PMC13632124; doi:10.1016/j.euros.2026.09.003)
Supplement: Supplementary file 1 [file mmc1.docx]

**Supplementary Methods**

*Modified JBI Case Series Critical Appraisal Checklist
for the systematic review "PSMA PET/CT kinetics during systemic therapy and radiotherapy in hormone-sensitive prostate cancer"*

**1. Rationale for using a modified version**

The standard JBI critical appraisal checklist for case series consists of 10 Yes/No/Unclear/Not applicable items and produces an overall qualitative appraisal but no per-domain rating on a low/moderate/serious scale. Quantitative poolwise synthesis of methodological quality is therefore not possible with the unmodified tool. Since our included studies are non-randomised observational studies reporting the longitudinal evolution of PSMA PET/CT parameters — a prognostic-style research question — we drew on both the JBI case series checklist and the Quality In Prognosis Studies (QUIPS) tool to construct a modified appraisal instrument that produces transparent per-domain ratings on a three-level (low / moderate / serious) scale, and an overall rating equivalent to the worst-rated domain. The resulting tool is explicit, reproducible, and aligned with international guidance (Cochrane Prognosis Methods Group; Hayden et al., Ann Intern Med 2013).

**2. Six risk-of-bias domains assessed**

Each included study is rated on the following six domains. Items 1–10 in the next section are the original JBI items mapped to these domains.

| **Domain** | **Description / rationale** |
| --- | --- |
| **1. Clarity of inclusion criteria (Study participation)** | Were inclusion/exclusion criteria for participants described clearly in the methods section? |
| **2. Standardised and reliable measurement of the condition (Study attrition – adapted)** | Was the PSMA PET/CT acquisition, reconstruction, and SUV measurement performed in a standardised and reproducible way, and was the same tracer used at baseline and follow-up? |
| **3. Consecutive or complete inclusion of participants** | Did the study include a consecutive or otherwise complete sample of participants meeting the criteria, or was the sample clearly non-consecutive / selective? |
| **4. Adequacy of reporting of clinical and demographic information** | Were relevant participant characteristics (age, disease stage, PSA, prior treatment, Gleason/ISUP score, lesion burden) adequately reported? |
| **5. Completeness of outcome reporting** | Were SU Vmax / volumetric outcomes reported with effect estimates, measures of variability (SD/IQR/range), and direction of change; were subgroup analyses clearly labelled? |
| **6. Appropriateness of statistical analysis** | Were statistical tests chosen appropriate for paired/within-patient comparisons and dependent observations; were confidence intervals or p-values reported where relevant? |

**3. Mapping of JBI items to the six domains**

The ten items of the standard JBI checklist for case series were grouped into the six domains above. The detailed item-by-item judgements for each included study are reported in Supplementary Table S1. The mapping is as follows:

| **JBI item (verbatim)** | **Mapped to domain** |
| --- | --- |
| JBI 1. Were there clear criteria for inclusion in the case series? | **Domain 1 — Clarity of inclusion criteria** |
| JBI 2. Was the condition measured in a standard, reliable way for all participants? | **Domain 2 — Standardised and reliable measurement** |
| JBI 3. Were valid methods used for identification of the condition for all participants? | **Domain 2 — Standardised and reliable measurement** |
| JBI 4. Did the case series have consecutive inclusion of participants? | **Domain 3 — Consecutive or complete inclusion** |
| JBI 5. Did the case series have complete inclusion of participants? | **Domain 3 — Consecutive or complete inclusion** |
| JBI 6. Was there clear reporting of the demographics of the participants? | **Domain 4 — Adequacy of reporting of clinical and demographic information** |
| JBI 7. Was there clear reporting of clinical information of the participants? | **Domain 4 — Adequacy of reporting of clinical and demographic information** |
| JBI 8. Were the outcomes or follow-up results of cases clearly reported? | **Domain 5 — Completeness of outcome reporting** |
| JBI 9. Was there clear reporting of the presenting site(s)/clinicodemographic information? | **Domain 4 — Adequacy of reporting of clinical and demographic information** |
| JBI 10. Was statistical analysis appropriate? | **Domain 6 — Appropriateness of statistical analysis** |

**4. Conversion rules — item responses → domain ratings**

| **Domain rating** | **Rule** |
| --- | --- |
| **Low risk of bias** | All relevant JBI items within the domain are answered 'Yes'. |
| **Moderate risk of bias** | One or more items within the domain are answered 'Unclear' AND no item is answered 'No'. |
| **Serious risk of bias** | One or more items within the domain are answered 'No'. |

**5. Overall risk-of-bias rating for each study**

The overall rating assigned to a study is the worst (highest-risk) rating obtained across its six domains. This mirrors the conservative approach advocated by the Cochrane Prognosis Methods Group when using QUIPS-based assessments, in which a single domain rated 'serious' is sufficient to lower confidence in the study's overall findings.

**6. Worked example**

As an illustration, Hoberück et al. (2020) was rated as having a serious risk of bias. Within Domain 3 (consecutive/complete inclusion) the JBI items concerning consecutive inclusion (item 4) and complete inclusion (item 5) were answered 'No' because the study selectively included patients meeting specific metabolic imaging criteria, resulting in a serious rating for that domain; since this is the worst domain rating, the overall rating for the study is serious.

**7. Item-by-item judgements for all 11 included studies**

*Y = Yes (item criterion met); N = No (item criterion not met); U = Unclear (insufficient information); NA = not applicable.*

| **JBI item** | **Malaspina 2023** | **Tseng 2022** | **Hoberück 2020** | **Afshar-Oromieh 2018** | **Onal 2020** | **Onal 2023** | **Onal 2024** | **Sheikh 2024** | **Zang 2025** | **Gawish 2023** | **Sadetski 2021** |
| --- | --- | --- | --- | --- | --- | --- | --- | --- | --- | --- | --- |
| **JBI 1 (Inclusion)** | **Y** | **Y** | **Y** | **Y** | **Y** | **Y** | **Y** | **Y** | **Y** | **Y** | **Y** |
| **JBI 2 (Standard measurement)** | **Y** | **Y** | **U** | **U** | **Y** | **Y** | **U** | **U** | **Y** | **U** | **U** |
| **JBI 3 (Valid identification)** | **Y** | **Y** | **U** | **U** | **Y** | **Y** | **U** | **U** | **Y** | **U** | **U** |
| **JBI 4 (Consecutive)** | **Y** | **U** | **N** | **N** | **U** | **U** | **U** | **N** | **U** | **U** | **U** |
| **JBI 5 (Complete inclusion)** | **Y** | **U** | **N** | **N** | **U** | **U** | **U** | **N** | **Y** | **U** | **U** |
| **JBI 6 (Demographics)** | **Y** | **Y** | **U** | **U** | **Y** | **Y** | **Y** | **U** | **N** | **U** | **Y** |
| **JBI 7 (Clinical info)** | **Y** | **U** | **U** | **U** | **U** | **U** | **U** | **U** | **Y** | **U** | **U** |
| **JBI 8 (Outcomes)** | **Y** | **U** | **U** | **U** | **U** | **U** | **U** | **U** | **U** | **U** | **U** |
| **JBI 9 (Site/clinicodemographic)** | **Y** | **Y** | **U** | **U** | **Y** | **Y** | **Y** | **U** | **Y** | **U** | **Y** |
| **JBI 10 (Statistical analysis)** | **Y** | **Y** | **U** | **Y** | **Y** | **U** | **Y** | **Y** | **U** | **U** | **N** |
| **OVERALL (worst domain)** | **Low** | **Moderate** | **Serious** | **Serious** | **Moderate** | **Moderate** | **Moderate** | **Serious** | **Serious** | **Moderate** | **Serious** |

**Legend (colour coding of judgements):** Y (Yes): criterion met (no shading); U (Unclear): insufficient information; N (No): criterion not met.

**Legend (colour coding of overall rating):** Low (light blue), Moderate (light yellow), Serious (light red).

**References**

• Munn Z, Barker TH, Moola S, et al. Methodological quality of case series studies: an introduction to the JBI critical appraisal tool. JBI Evid Synth 2020;18:2127–33.

• Aromataris E, Munn Z (Editors). JBI Manual for Evidence Synthesis. JBI, 2020. Available from https://synthesismanual.jbi.global.

• Hayden JA, van der Windt DA, Cartwright JL, Côté P, Bombardier C. Assessing bias in studies of prognostic factors. Ann Intern Med 2013;158:280–6.

• Riley RD, Moons KGM, Snell KIE, et al. A guide to systematic review and meta-analysis of prognostic factor studies. BMJ 2019;364:k4597.

• Forbes A, Turner S, Grooten WJA, et al. Elaborating on the assessment of the risk of bias in prognostic factor studies: a methodological review. BMC Med Res Methodol 2019;19:Article 23.

• Cochrane Prognosis Methods Group. Resources for authors of Cochrane prognosis reviews. Available from https://methods.cochrane.org/prognosis.
