# Supplementary material for: PSMA PET/CT Kinetics During Systemic Therapy and Radiotherapy in Hormone-sensitive Prostate Cancer: A Systematic Review
Source: Eur Urol Open Sci. 2026 Sep 21;93:14–25. doi: 10.1016/j.euros.2026.09.003 (PMC13632124; doi:10.1016/j.euros.2026.09.003)
Supplement: Supplementary file 2 [file mmc2.docx]

Database(s): MEDLINE (via PubMed)

Filters: No restrictions regarding the language or date of the publication were used. The search strategy was executed on 2025/12/08.

| # | search syntax | number of hits |
| --- | --- | --- |
| 1 | "prostatic neoplasms"[MeSH Terms] OR ("prostat*"[Title/Abstract] AND ("cancer"[Title/Abstract] OR "carcinoma"[Title/Abstract] OR "adenocarcinoma"[Title/Abstract] OR "malignan*"[Title/Abstract] OR "tumor"[Title/Abstract] OR "tumour"[Title/Abstract] OR "neoplas*"[Title/Abstract])) | 235,246 |
| **2** | "PSMA-PET"[All Fields] OR (("Prostate-Specific Membrane Antigen"[All Fields] OR PSMA[TIAB]) AND ("Positron Emission Tomography"[All Fields] OR "PET"[All Fields] OR PET/CT[All Fields] OR PET/MRI[All Fields])) OR "Ga-68 PSMA"[All Fields] OR "68Ga-PSMA"[All Fields] OR "Ga-PSMA"[All Fields] OR "Gallium-68 PSMA"[All Fields] OR "68Gallium-PSMA"[All Fields] OR "Gallium-PSMA"[All Fields] OR "F-18-PSMA"[All Fields] OR "18F-PSMA"[All Fields] OR "F-PSMA"[All Fields] OR "F-flotufolastat"[All Fields] OR "F-Piflufolastat"[All Fields] | 5,645 |
| 3 | "standardi*ed uptake value"[Title/Abstract] OR "SUV"[Title/Abstract] OR "SUVmax"[Title/Abstract] OR "SUVmean"[Title/Abstract] OR "total tumo*r volume"[Title/Abstract] OR "TTV"[Title/Abstract] OR "PSMA score"[Title/Abstract] OR "PSMA tumor volume"[Title/Abstract] OR "PSMA-TV"[Title/Abstract] OR "molecular tumor volume"[Title/Abstract] OR "MTV"[Title/Abstract] OR "total lesion PSMA"[Title/Abstract] OR "TLP"[Title/Abstract] OR "tracer uptake"[Title/Abstract] OR "PSMA uptake"[Title/Abstract] OR "maximum uptake"[Title/Abstract] OR "avidity"[ Title/Abstract] OR "expression"[ Title/Abstract] OR "miPSMA"[Title/Abstract] OR "PSMA-RADS"[Title/Abstract] OR "RECIP"[Title/Abstract] OR "aPERCIST"[Title/Abstract] OR "PPP"[Title/Abstract] | 2,791,116 |
| 4 | "flare"[Title/Abstract] OR "response"[Title/Abstract] OR "reduc*"[Title/Abstract] OR "decrease*"[Title/Abstract] OR "decline"[Title/Abstract] OR "remission"[Title/Abstract] OR "change"[Title/Abstract] OR "delta"[Title/Abstract] OR "before and after"[Title/Abstract] OR "pre and post"[Title/Abstract] | 10,370,458 |
| 5 | #1 #2 #3 #4 | 563 |

Database(s): Embase (via Embase.com)

Filters: No restrictions regarding the language or date of the publication were used. The search strategy was executed on 2025/12/08.

| # | search syntax | number of hits |
| --- | --- | --- |
| 1 | 'prostate tumor'/exp OR ('prostat*':ti,ab,kw AND ('cancer':ti,ab,kw OR 'carcinoma':ti,ab,kw OR 'adenocarcinoma':ti,ab,kw OR 'malignan*':ti,ab,kw OR 'tumor':ti,ab,kw OR 'tumour':ti,ab,kw OR 'neoplas*':ti,ab,kw)) | 405,692 |
| **2** | 'psma-pet' OR (('prostate-specific membrane antigen'/exp OR 'prostate-specific membrane antigen' OR 'psma':ti,ab,kw) AND ('positron emission tomography'/exp OR 'positron emission tomography' OR 'pet'/exp OR 'pet' OR 'pet/ct' OR 'pet/mri')) OR 'ga-68 psma' OR '68ga-psma' OR 'ga-psma' OR 'gallium-68 psma' OR '68gallium-psma' OR 'gallium-psma' OR 'f-18-psma' OR '18f-psma' OR 'f-psma' OR 'f-flotufolastat' OR 'f-piflufolastat' | 13,163 |
| 3 | 'standardi*ed uptake value':ti,ab,kw OR 'suv':ti,ab,kw OR 'suvmax':ti,ab,kw OR 'suvmean':ti,ab,kw OR 'total tumo*r volume':ti,ab,kw OR 'ttv':ti,ab,kw OR 'psma score':ti,ab,kw OR 'psma tumor volume':ti,ab,kw OR 'psma-tv':ti,ab,kw OR 'molecular tumor volume':ti,ab,kw OR 'mtv':ti,ab,kw OR 'total lesion psma':ti,ab,kw OR 'tlp':ti,ab,kw OR 'tracer uptake':ti,ab,kw OR 'psma uptake':ti,ab,kw OR 'maximum uptake':ti,ab,kw OR 'avidity':ti,ab,kw OR 'expression':ti,ab,kw OR 'mipsma':ti,ab,kw OR 'psma-rads':ti,ab,kw OR 'recip':ti,ab,kw OR 'apercist':ti,ab,kw OR 'ppp':ti,ab,kw | 3,651,690 |
| 4 | 'flare':ti,ab,kw OR 'response':ti,ab,kw OR 'reduc*':ti,ab,kw OR 'decrease*':ti,ab,kw OR 'decline':ti,ab,kw OR 'remission':ti,ab,kw OR 'change':ti,ab,kw OR 'delta':ti,ab,kw OR 'before and after':ti,ab,kw OR 'pre and post':ti,ab,kw | 13,693,411 |
| 5 | #1 AND #2 AND #3 AND #4 AND [embase]/lim | 1,508 |

Database(s): Scopus

Filters: No restrictions regarding the language or date of the publication were used. The search strategy was executed on 2025/12/08.

| # | search syntax | number of hits |
| --- | --- | --- |
| 1 | INDEXTERMS ( "prostatic neoplasms" ) OR ( TITLE-ABS ( prostat* ) AND ( TITLE-ABS ( cancer ) OR TITLE-ABS ( carcinoma ) OR TITLE-ABS ( adenocarcinoma ) OR TITLE-ABS ( malignan* ) OR TITLE-ABS ( tumor ) OR TITLE-ABS ( tumour ) OR TITLE-ABS ( neoplas* ) ) ) | 235,246 |
| **2** | ALL("PSMA-PET") OR ((ALL("Prostate-Specific Membrane Antigen") OR TITLE-ABS("PSMA")) AND (ALL("Positron Emission Tomography") OR ALL("PET") OR ALL("PET/CT") OR ALL("PET/MRI"))) OR ALL("Ga-68 PSMA") OR ALL("68Ga-PSMA") OR ALL("Ga-PSMA") OR ALL("Gallium-68 PSMA") OR ALL("68Gallium-PSMA") OR ALL("Gallium-PSMA") OR ALL("F-18-PSMA") OR ALL("18F-PSMA") OR ALL("F-PSMA") OR ALL("F-flotufolastat") OR ALL("F-Piflufolastat") | 18,358 |
| 3 | TITLE-ABS("standardied uptake value") OR TITLE-ABS("SUV") OR TITLE-ABS("SUVmax") OR TITLE-ABS("SUVmean") OR TITLE-ABS("total tumor volume") OR TITLE-ABS("TTV") OR TITLE-ABS("PSMA score") OR TITLE-ABS("PSMA tumor volume") OR TITLE-ABS("PSMA-TV") OR TITLE-ABS("molecular tumor volume") OR TITLE-ABS("MTV") OR TITLE-ABS("total lesion PSMA") OR TITLE-ABS("TLP") OR TITLE-ABS("tracer uptake") OR TITLE-ABS("PSMA uptake") OR TITLE-ABS("maximum uptake") OR TITLE-ABS("avidity") OR TITLE-ABS("expression") OR TITLE-ABS("miPSMA") OR TITLE-ABS("PSMA-RADS") OR TITLE-ABS("RECIP") OR TITLE-ABS("aPERCIST") OR TITLE-ABS("PPP") | 3,902,198 |
| 4 | TITLE-ABS ( "flare" ) OR TITLE-ABS ( "response" ) OR TITLE-ABS ( "reduc*" ) OR TITLE-ABS ( "decrease*" ) OR TITLE-ABS ( "decline" ) OR TITLE-ABS ( "remission" ) OR TITLE-ABS ( "change" ) OR TITLE-ABS ( "delta" ) OR TITLE-ABS ( "before and after" ) OR TITLE-ABS ( "pre and post" ) | 27,863,259 |
| 5 | (INDEXTERMS ( "prostatic neoplasms" ) OR ( TITLE-ABS ( prostat* ) AND ( TITLE-ABS ( cancer ) OR TITLE-ABS ( carcinoma ) OR TITLE-ABS ( adenocarcinoma ) OR TITLE-ABS ( malignan* ) OR TITLE-ABS ( tumor ) OR TITLE-ABS ( tumour ) OR TITLE-ABS ( neoplas* ) ) )) AND (ALL("PSMA-PET") OR ((ALL("Prostate-Specific Membrane Antigen") OR TITLE-ABS("PSMA")) AND (ALL("Positron Emission Tomography") OR ALL("PET") OR ALL("PET/CT") OR ALL("PET/MRI"))) OR ALL("Ga-68 PSMA") OR ALL("68Ga-PSMA") OR ALL("Ga-PSMA") OR ALL("Gallium-68 PSMA") OR ALL("68Gallium-PSMA") OR ALL("Gallium-PSMA") OR ALL("F-18-PSMA") OR ALL("18F-PSMA") OR ALL("F-PSMA") OR ALL("F-flotufolastat") OR ALL("F-Piflufolastat")) AND (TITLE-ABS("standardied uptake value") OR TITLE-ABS("SUV") OR TITLE-ABS("SUVmax") OR TITLE-ABS("SUVmean") OR TITLE-ABS("total tumor volume") OR TITLE-ABS("TTV") OR TITLE-ABS("PSMA score") OR TITLE-ABS("PSMA tumor volume") OR TITLE-ABS("PSMA-TV") OR TITLE-ABS("molecular tumor volume") OR TITLE-ABS("MTV") OR TITLE-ABS("total lesion PSMA") OR TITLE-ABS("TLP") OR TITLE-ABS("tracer uptake") OR TITLE-ABS("PSMA uptake") OR TITLE-ABS("maximum uptake") OR TITLE-ABS("avidity") OR TITLE-ABS("expression") OR TITLE-ABS("miPSMA") OR TITLE-ABS("PSMA-RADS") OR TITLE-ABS("RECIP") OR TITLE-ABS("aPERCIST") OR TITLE-ABS("PPP")) AND (TITLE-ABS ( "flare" ) OR TITLE-ABS ( "response" ) OR TITLE-ABS ( "reduc*" ) OR TITLE-ABS ( "decrease*" ) OR TITLE-ABS ( "decline" ) OR TITLE-ABS ( "remission" ) OR TITLE-ABS ( "change" ) OR TITLE-ABS ( "delta" ) OR TITLE-ABS ( "before and after" ) OR TITLE-ABS ( "pre and post" )) | 904 |
